# Side-by-Side Comparison of Gene-Based Smallpox Vaccine with MVA in Nonhuman Primates

**DOI:** 10.1371/journal.pone.0042353

**Published:** 2012-07-31

**Authors:** Joseph W. Golden, Matthew Josleyn, Eric M. Mucker, Chien-Fu Hung, Peter T. Loudon, T. C. Wu, Jay W. Hooper

**Affiliations:** 1 Department of Molecular Virology, Virology Division, United States Army Medical Research Institute of Infectious Diseases, Fort Detrick, Maryland, United States of America; 2 Department of Viral Therapeutics, Virology Division, United States Army Medical Research Institute of Infectious Diseases, Fort Detrick, Maryland, United States of America; 3 Department of Pathology, Johns Hopkins Medical Institutions, Baltimore, Maryland, United States of America; 4 Pfizer, Sandwich Laboratories, Sandwich, Kent, United Kingdom; Public Health Agency of Canada, Canada

## Abstract

Orthopoxviruses remain a threat as biological weapons and zoonoses. The licensed live-virus vaccine is associated with serious health risks, making its general usage unacceptable. Attenuated vaccines are being developed as alternatives, the most advanced of which is modified-vaccinia virus Ankara (MVA). We previously developed a gene-based vaccine, termed 4pox, which targets four orthopoxvirus antigens, A33, B5, A27 and L1. This vaccine protects mice and non-human primates from lethal orthopoxvirus disease. Here, we investigated the capacity of the molecular adjuvants GM-CSF and *Escherichia coli* heat-labile enterotoxin (LT) to enhance the efficacy of the 4pox gene-based vaccine. Both adjuvants significantly increased protective antibody responses in mice. We directly compared the 4pox plus LT vaccine against MVA in a monkeypox virus (MPXV) nonhuman primate (NHP) challenge model. NHPs were vaccinated twice with MVA by intramuscular injection or the 4pox/LT vaccine delivered using a disposable gene gun device. As a positive control, one NHP was vaccinated with ACAM2000. NHPs vaccinated with each vaccine developed anti-orthopoxvirus antibody responses, including those against the 4pox antigens. After MPXV intravenous challenge, all control NHPs developed severe disease, while the ACAM2000 vaccinated animal was well protected. All NHPs vaccinated with MVA were protected from lethality, but three of five developed severe disease and all animals shed virus. All five NHPs vaccinated with 4pox/LT survived and only one developed severe disease. None of the 4pox/LT-vaccinated animals shed virus. Our findings show, for the first time, that a subunit orthopoxvirus vaccine delivered by the same schedule can provide a degree of protection at least as high as that of MVA.

## Introduction

Naturally occurring smallpox was eradicated in the late 20^th^ century through a coordinated worldwide vaccination campaign [1]. Nevertheless, variola virus (VARV), the causative agent of smallpox, or a genetically derived pathogenic orthopoxvirus, pose a biological weapons threat that has prompted the need for continued vigilance to prevent the accidental or purposeful reemergence of this family of viruses in the human population. Other orthopoxviruses are also a threat to public health in the form of emerging zoonoses [2]. These include human monkeypox virus (MPXV), cowpox virus (CPXV), and a variety of vaccinia virus (VACV)-like viruses circulating throughout the world [3], [4], [5], [6]. These viruses, though not as virulent as VARV, still cause significant morbidity and occasional mortality in places such as central Africa, Eurasia and South America. The outbreak of MPXV in the Midwestern United States has also shown that these zoonoses are a worldwide health threat [7]. Whereas protecting groups of the population against emerging zoonoses is relatively unappreciated, the desire to defend against a potential biological weapons attack has led to a renewed effort to develop and stockpile orthopoxvirus vaccines and therapeutics [8].

The original VACV calf-lymph produced smallpox vaccine is no longer manufactured; however, a second-generation cell-culture-derived version of the vaccine, ACAM2000®, has been produced [9]. This vaccine, just as the calf-lymph produced vaccine [10], is associated with significant, even life threatening health risks, including myocarditis [11], [12]. Accordingly, it is contraindicated for persons suffering from, or living with persons suffering from, a variety of conditions including the relatively common condition eczema [12]. The health risks associated with the live-virus vaccine have prompted the development of third-generation attenuated vaccines. Currently, modified vaccinia Ankara (MVA) is the most advanced [13], but other attenuated vaccines, including Lc16m8, are under development [14]. A version of MVA called IMVAMUNE®, produced by Barvarian Nordic, has been fast-tracked for licensure by the US Food and Drug Administration (FDA) [15]. While these attenuated vaccines have a significantly improved safety profile, they have lower immunogenicity and typically require two doses for full protection [13], [14]. Similar to ACAM2000®, the protective components of these vaccines are undefined, as the viruses express hundreds of encoded gene products [16], most of which are not likely to contribute to protection.

As highly-defined alternatives to live-virus vaccines, several groups have independently developed protein or gene-based vaccines against orthopoxviruses (for a review see [17]). To date, most of these vaccines have targeted molecules, individually or in combination, located on the mature virion (MV) and/or the enveloped virion (EV). The orthopoxvirus MV and EV are biologically distinct forms of the virus, and both are infectious [45]. In addition to the structural protein targets, the non-structural protein type I interferon (IFN)-binding molecule (B18/B19) has been shown to be a protective immunogen in certain models [18]. While it has been well established by several independent groups that antibodies are the most critical component for vaccine-induced protection against orthopoxviruses [19], [20], [21], [22], vaccines targeting T-cell epitopes have also been shown to offer some protection [23], [24].

Because the MV and EV particles are biologically and immunologically distinct [25], targeting molecules present on both forms confers the best protection [26], [27], [28]. Accordingly, many experimental subunit vaccines under development target various combinations of molecules present on both particles. For example, many of these vaccines have targeted the MV immunogens L1 and A27 combined with the EV immunogens A33 and B5. Vaccines comprised of this MV/EV combination of immunogens have shown efficacy in a multitude of animal models, including VACV in mice [26], [27], [28] and MPXV in NHPs [29], [30], [31], [32], [33]. A subset of these multicomponent vaccines contains only the L1, A27, A33, and B5 targets, which we have called 4pox [28], [31], [34]. The 4pox vaccine delivered by various technologies has been shown to protect in various animal models, including the VACV intranasal murine model and the MPXV intravenous NHP model [28], [31], [32], [34], [35], [36].

While no molecular smallpox vaccine has been tested for immunogenicity in humans, it is possible to compare molecular smallpox vaccines with live-virus smallpox vaccines that have been tested in human. Here, we conducted a direct comparison of a modified version of the 4pox smallpox DNA vaccine delivered by particle mediated epidermal delivery (gene gun) with MVA delivered intramuscularly. These vaccines, administered twice at a 1-month interval, were compared in nonhuman primates for a capacity to elicit antibody responses against the L1, A27, A33, and B5 targets, and to protect against lethal monkeypox after an intravenous challenge.

## Materials and Methods

### Ethics statement

Research was conducted in compliance with the Animal Welfare Act and other federal statutes and regulations relating to animals and experiments involving animals and adheres to principles stated in the Guide for the Care and Use of Laboratory Animals, National Research Council, 1996. The facilities where this research was conducted are fully accredited by the Association for Assessment and Accreditation of Laboratory Animal Care International. All animal experiments were approved by USAMRIID's Institutional Animal Care and Use Committee (approval ID AP-06-047, AP-09-044).

### Cells and viruses

VACV Connaught vaccine strain (derived from the New York City Board of Health strain), VACV strain WR (ATCC VR-1354), and VACV strain IHD-J were maintained in VERO cell (ATCC CRL-1587) monolayers grown in Eagle minimal essential medium containing 5% heat-inactivated fetal bovine serum (FBS), 1% antibiotics (100 U/ml penicillin, 100 µg/ml of streptomycin, and 50 µg/ml of gentamicin), 10 mM HEPEs (cEMEM). COS-7 (COS) cells (ATCC CRL-1651) were used for transient expression experiments. BSC-1 cells (ATCC CCL-26) were used for plaque-reduction neutralization assays (PRNT) and EV spread inhibition assays. Both BSC-1 and COS cells were maintained in cEMEM. MPXV strain Zaire-79 used in challenge experiments was kindly provided by Dr. John Huggins.

### Plasmids

The 4pox genes were synthesized *de novo* after having been optimized for mRNA stability and codon usage in human cells (GeneArt; Burlingame, CA)). Generation of the L1R plasmid was done essentially as described previously [36]. A33R, B5R, and A27R were also optimized for expression, including the addition of a kozak sequence on the 5′ untranslated region of the genes. To construct the pWRG/A33Rkopt, pWRG/B5Rkopt and pWRG/A27kopt, open reading frames from the *de novo* synthesized genes were cloned into the NotI and BglII sites of the pWRG vector. Each construct was confirmed by sequencing and expression using monoclonal and polyclonal antibodies before use in vaccine studies (data not shown).

pPJV2012(DEI-LT) (expressing *E. coli* heat labile enterotoxin) and pPML7805(GM-CSF) (expressing murine GM-CSF) were provided by Pfizer and have been tested in other systems [37], [38].

### DNA vaccination of mice with research gene-gun

The procedure for preparing cartridges for the research gene gun and vaccinating mice was described previously [27], [39]. All mice were at least 7–9 weeks old at the start of vaccination. All mouse work performed in this study was approved by the USAMRIID Institutional Animal Care and Use Committee (IACUC).

### DNA vaccination of nonhuman primates with single-use, hand-held ND10 device

Rhesus macaques (*Maccaca mulatta*) were obtained from an approved vendor. The sex and weight of each animal is provided in **Table 1**. All animals were negative for evidence of previous exposure to orthopoxviruses as measured by ELISA (data not shown). For DNA vaccinations, the NHP were vaccinated using single-use, hand-held ND10 devices manufactured at Pfizer. ND10 devices are a version of gene-gun designed for use in clinical trials. ND10 consist of a helium gas microcylinder, an expansion chamber with a cassette containing the DNA vaccine-coated gold particles, nozzle, and actuation button. Vaccination was done on the upper arm and alternate arms were used for the prime and boosting vaccination. Each animal received four administrations (four devices) per vaccination of the indicated vaccine, 4pox with adjuvant or empty vector. This resulted in a total vaccine dose of 8 µg of total DNA/dose. All NHP work performed in this study was approved by the USAMRIID IACUC.

**Table 1:** Results of MPXV i.v. challenge.

Monkey ID	Sex/weight (kg)	Group	Vaccine	Peak lesion#	Disease	Day of Death	Shedding (pfu/ml throat swab)^1^	Blood viremia (genomes/ml)^1^
0600	F/3.9	1	4pox/LT	23	moderate	Survived	<	553
1104	F/4.5	1	4pox/LT	18	moderate	Survived	<	6,620
0014	F/4.6	1	4pox/LT	12	moderate	Survived	<	900
5854	F/5.1	1	4pox/LT	64	moderate	Survived	<	770
1112	F/5.1	1	4pox/LT	132	severe	Survived	<	9,520
3868	F/4.3	2	Neg Control	>500	grave	9	40,500	12,965,000
1116	F/4.5	2	Neg Control	>500	grave	12	65,250	12,185,000
0290	F/4.6	2	Neg Control	350	severe	Survived	81,250	824,500
0050	F/5.0	2	Neg Control	>500	grave	11	20,750	4,945,000
3907	M/6.9	2	Neg Control	254	grave	7	25,000	3,208,300
3890	F/4.4	3	MVA	48	moderate	Survived	500	8,125
0060	F/4.4	3	MVA	118	severe	Survived	2,100	2,550
0470	F/4.7	3	MVA	11	moderate	Survived	6,725	10,445
1838	F/4.7	3	MVA	239	severe	Survived	1,750	9,465
9923	M/7.1	3	MVA	269	severe	Survived	3,000	304,950
3802	F/4.8	4	ACAM2000	4	mild	Survived	<	<

<limit of detection 100 pfu/ml throat swab suspension.

1 Mean of two highest consecutive time points.

### Vaccination of NHP with live-virus vaccines

ACAM2000 Lot VV04-003A was provided by USAMRIID's Division of Medicine. NHPs were vaccinated using a bifurcated needle (30 pricks) according to the instructions on the package insert. ACAM2000 was administered as a single dose. MVA (NR-70, Lot 460301DA) was obtained from BEI Resources (Manassas, VA). The lyophilized MVA product was reconstituted in water as instructed on label. Animals were vaccinated intramuscularly in the deltoid with 1×10^8^ pfu of the vaccine per dose. MVA was administered as a two-dose, 1-month interval, vaccine.

### ELISAs

ELISA using VACV histidine-tagged antigens L1 (300 ng/well), A33 (50 ng/well), B5 (50 ng/well) and A27 (50 ng/well), produced in *Escherichia coli* or, in the case of B5, baby hamster kidney cells, has been described in detail previously [31]. As described previously, an irrelevant his-tagged protein purified from *E. coli* (BotN) was used as a negative control antigen. Endpoint titers were calculated as the highest dilution with an absorbance value greater than the mean absorbance value from negative control sera plus three standard deviations. To determine the IgG subclass, secondary antibodies (conjugated to HRP) against IgG1 (1∶1000) and IgG2a (1∶1000) (Bethyl laboratories; Montgomery, TX) were incubated in duplicate plates. The ratio of IgG1/IgG2a was calculated and graphed.

VACV-infected-cell lysate ELISAs were performed as described [31], [36]. VACV strain WR infected-cell lysate or mock antigen was diluted 1∶50 in PBS and added to the wells of a 96-well high-binding ELISA plate (Corning; Corning, NY) and dried overnight. Mock antigen consisted of lysates made from uninfected cells processed in an identical manner to infected-cells.

### Neutralization assays

The plaque-reduction and neutralization assay (PRNT) has been described previously [27], [39]. Briefly, VACV strain IHD-J or MPXV strain Zaire-79 were diluted in cEMEM to give ∼250 pfu/ml. Aliquots of this viral suspension were incubated with an equal volume of serum diluted in cEMEM for 1 h at 37°C. Samples were then adsorbed to confluent BSC-1 cell monolayers in 6-well plates for 1 h in a 37°C 5% CO_2_ incubator. After adsorption, a 2 ml semisolid overlay (Earle's basal minimal essential medium, 1.5% methyl cellulose, 5% heat inactivated FBS, antibiotics (100 U/ml penicillin, 100 µg/ml of streptomycin, and 50 µg/ml of gentamicin) was added to each well. After 3 days (VACV) or 6 days (MPXV) in a 37°C 5% CO_2_ incubator, cell monolayers were stained by adding 1 ml/well of a staining solution (3% crystal violet and 15% ethanol in H_2_O) overnight. Monolayers were rinsed with water and the plaques were counted. The percent neutralization was calculated relative to the number of plaques in the absence of antibody. Titers represent the reciprocal of the highest dilution resulting in a 50% reduction in the number of plaques in the absence of antibody.

EV neutralization was preformed as previously reported with slight modifications [40], [41]. Briefly, fresh EV particles (75–100 pfu) were incubated for 1 h with the indicate sera (diluted twofold starting at a 1∶40) in the presence or absence of 5% human complement (Sigma) in 200 µl total volume. After incubation, the mixture of 180 µl was adsorbed for 1 h on BSC-1 cell monolayers in 6-well plates. Warm PBS was used to wash away unbound virus and 2 ml of semisolid overlay (see above) was added to each well. All samples included the anti-MV monoclonal antibody, MAb-10F5 (1∶100) which targets the L1 molecule [27], [42]. After 4 days plaques were stained with crystal violet as described above. Plaques were counted and the percent neutralization was calculated relative to the number of plaques in the absence of anti-EV antibody or in the absence of anti-EV antibody, but in the presence of complement. Titers represent the reciprocal of the highest dilution resulting in a 50% reduction in the number of plaques. Each experiment was performed in duplicate.

### Intranasal VACV mouse challenges

Mice were anesthetized and weighed before intranasal administration of 50 µl of PBS containing 2×10^6^ pfu (three times LD_50_) of VACV strain IHD-J using a plastic pipette tip. Subsequently, mice were observed for signs of disease and weighed daily for 14 days. Mice were provided food and water *ad libitum*. Every effort was made to minimize suffering. Based on predetermined criteria (e.g., >30% body weight) moribund mice were euthanized in accordance with the 2007 Report of the American Veterinary Medical Association Panel on Euthanasia.

### Non-human primate MPXV challenge

NHPs were challenged with MPXV strain Zaire-79 7 weeks after the last vaccination. Animals were anesthetized by an i.m. injection with ketamine HCL at approximately 10–30 mg/kg and inoculated through the saphenous vein using a 22–25 gauge needle with 1 ml of medium containing 2×10^7^ pfu/ml of MPXV strain Zaire-79. NHPs were monitored for disease at least twice daily. Lesions counts, throat swabs, temperature, weight and blood collection were monitored on days -6, -3, 0, 1, 2, 3, 6, 9, 12, 15, 18, 21 and 28. Hematological values were generated from fresh, whole blood using a Coulter AcT Series analyzer (Coulter Corp.; Miami, FL). Animals that developed monkeypox were administered meloxicam at 0.3 mg/kg/day as analgesia. Oral rehydration solution was provided via a bottle attached to the cage, and high moisture fruits were provided. Every effort was made to minimize suffering. Animals meeting predetermined disease criteria were euthanized in accordance with the 2007 Report of the American Veterinary Medical Association Panel on Euthanasia.

### Detecting infectious virus from throat swabs and whole blood

Throat swabs were taken at each time point using a sterile cotton tip applicator and swirling 10 times in the back of the throat. Cotton tips were broken off in sterile tubes and resuspended in 400 µl of cEMEM. Samples were sonicated for 30 s. Throat swab suspensions and whole blood were evaluated for the presence of infectious virus by plaque assay. The lowest dilution tested was 1∶100. 200 µl of inoculum was adsorbed on confluent monolayers of BSC cells for 1 h with rocking ∼15 m. After the adsorption, 2 ml of a 1∶1 mixture of 3% methocellulose and 2× EBME were added to each well. Plates were incubated for 5 days at 37°C and then stained with 1.5% crystal violet stain containing 30% formalin to visualize plaques.

### Quantitative PCR

Nucleic acid extractions were completed using the Qiagen QIAmp DNA Blood Mini kit (catalog# 51106) according to the manufacturer's instructions (Qiagen, Valencia CA.). Briefly, 100 µl of EDTA blood was extracted and eluted into 100 µl of buffer AE (provided by manufacturer). QPCR reactions were performed according to Kulesh, et al. [43], with the following exceptions: the Roche LightCycler 480 was used for the assay and analyzed with the LightCycler 480 SW 1.5 software (Roche Applied Sciences, Indianapolis, IN). The cycling parameters were as follows: 2 min at 94°C for 1 cycle (4.4 C/s); 10 sec at 94°C (4.4 C/s) and 20 sec at 60°C (2.2 C/s) for 45 cycles; 30 sec at 40°C (2.2C/s) for one cycle. Values were generated using 2^nd^ derivate maximum (absolute quantitation), averaged, and multiplied by 200 to yield viral genomes per ml of blood. The limit of quantitation (LOQ) and limit of detection (LOD) for the assay was 50 genomes per 5 µl (or 10000 genomes/ml) and 2.5 genomes per 5 µl (500 genomes/ml).

### Statistics

Repeated measures ANOVA of weight among groups with stepdown Bonferroni adjustment for pairwise comparisons were performed to determine if there were significant differences in weight loss in mice exposed to VACV. For mice, Fisher's Exact tests were used to compare survival rates among groups. For NHP, Fisher's exact test with step-down bootstrap adjustment were used to compare survival rates. Kaplan Meier survival analysis and log-rank tests with stepdown Bonferroni adjustment were used to compare survival curves. When evaluating differences in GMT between groups, t-tests were performed with step-down Bonferroni adjustment for multiple comparisons. ANOVA with day as the repeated measure were used to evaluate lesion counts, throat swab viremia, and Q-PCR. T-tests were used to detect differences between groups on each day. Significance was set at a *p*-value >0.05.

## Results

### Antibody responses against 4pox DNA vaccine immunogens were enhanced when DEI GM-CSF or DEI LT were included as adjuvants

**Figure 1:**
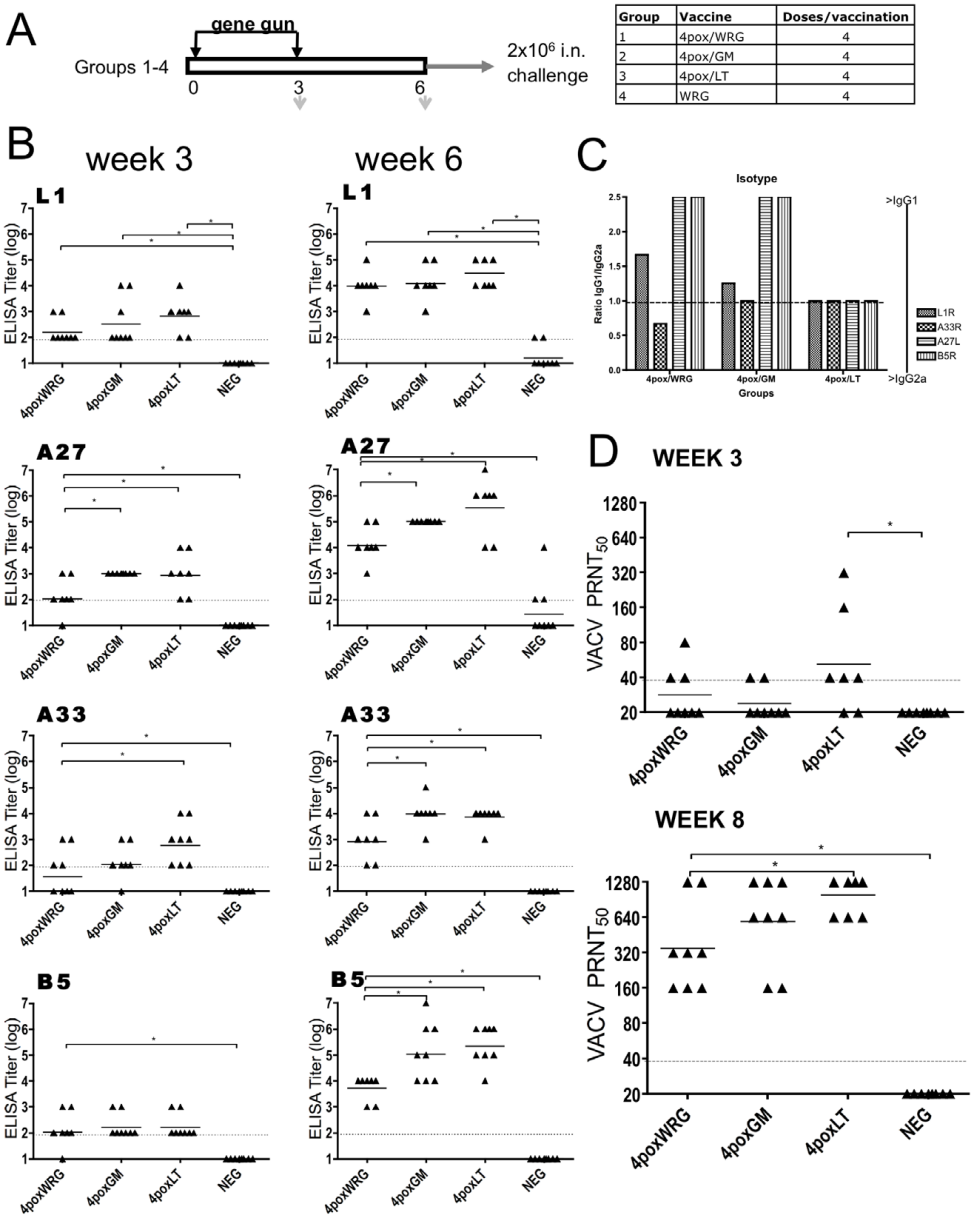
Antibody responses against the 4pox vaccine targets in vaccinated mice. **A**. Schematic showing the vaccination protocol. Mice were vaccinated by gene-gun twice at 3-week intervals with the 4pox vaccine and empty vector (4pox/WRG), 4pox plus GM-CSF (4pox/GM-CSF) or LT (4pox/LT) or a negative control vaccine containing irrelevant DNA. All mice were vaccinated with four cartridges per vaccination equaling ∼2 µg DNA/vaccination. Bleed dates are indicated by the down arrows. **B**. Purified 4pox antigens (L1, A33, B5, and A27) were plated in 96-well plates. Sera from mice vaccinated on week 3 (prime) or week 6 (boost) with the indicated vaccines were serially diluted tenfold (from 1∶100) and incubated with purified protein. Endpoint titers were calculated as described in the materials and methods. Data were plotted a scatter plot showing the distribution of the titer along with a horizontal line indicating the geometric mean titer. **C**. Pooled sera from vaccinated mice were serially diluted tenfold. Dilutions were incubated with duplicate plates containing the 4pox antigens. Secondary anti-mouse antibodies specific for either IgG1 or IgG2a were then incubated with the samples. Endpoint titers for each secondary antibody were calculated as in part B. The ratio of IgG1 to IgG2a was then determined and plotted. **D**. Sera from mice vaccinated on week 3 (prime) or week 6 (boost) with the indicated vaccines were serially diluted twofold and incubated with VACV strain IHD-J. 50% neutralization titers were calculated relative to the plaque count for virus that was not incubated with serum. Data were plotted as a mean titer for each group +/− standard deviation. All statistics were calculated as described in the material and methods and are denoted by an asterisks sign as well as a line showing which groups are being compared.

To determine if it was possible to enhance the immune response to the 4pox DNA vaccine using DNA encoded immunostimulatory (DEI) molecules, we examined the extent to which granulocyte-macrophage colony-stimulating factor (GM-CSF) or *E. coli* heat-labile enterotoxin (LT) could enhance the immune response against the 4pox vaccine targets. Groups of eight mice were vaccinated twice at 3-week intervals with either the 4pox vaccine with empty vector (4poxWRG), or GM-CSF (4pox/GM), or LT (4pox/LT) (**Fig. 1A**). Mice were also vaccinated with a negative control DNA vaccine (WRG). Antibody responses after vaccination were evaluated by immunogen-specific ELISAs. After the initial priming vaccination, antibody responses were detected against each target in most animals (**Fig. 1B**). With the exception of B5, responses in groups receiving the 4pox vaccine, plus adjuvants were higher after the prime (week 3). The antibody response against A27 was significantly higher (p<0.05) in groups receiving GM-CSF and LT compared to those not receiving DEI adjuvant. The response against A33 was significantly higher (p<0.05) in groups receiving LT, but not GM-CSF. L1 responses were not significantly greater than the 4pox/WRG group after the prime. Three weeks after the boost (week 6), the anamnestic responses markedly increased for each target (**Fig. 1B**). Both GM-CSF and LT significantly enhanced the antibody responses against A27, A33, and B5. This was most dramatic for the A27 and B5 targets, where GMTs increased greater than a log. The response to L1 was not significantly enhanced by the DEI.

Administration of DNA vaccines via gene-gun predominantly produce a Th2 response characterized by a higher IgG1 subclass antibody response versus the IgG2a response [44]. We examined the antibody subclass responses in mice vaccinated with the 4pox vaccine and found that inclusion of LT invoked a balanced Th1/Th2 responses against all four targets as determined by the ratio of IgG1 (Th2) and IgG2a (Th1). In contrast, GM-CSF induced a balanced A33 response, but did not alter the subclass response of the other vaccine components compared to the unadjuvanted vaccine (**Fig. 1C**).

We investigated the functionality of the antibody responses by performing PRNT to measure neutralizing antibodies. After the initial vaccination (week 3), only mice vaccinated with 4pox/LT had statistically significant neutralizing responses compared to negative control animals (p = 0.0004) (**Fig. 1D**). However, after the boost (week 6), all groups receiving the 4pox vaccine had neutralizing antibody responses as measure by PRNT regardless of the addition of adjuvant. Animals vaccinated with 4pox/LT had significantly higher responses compared to the 4poxWRG vaccine (GMT = 987 versus GMT = 349, p = 0.0079). The PRNT GMT was slightly higher in the 4pox/GM animals versus the 4poxWRG animals, but the difference was not statistically significant. Overall, our findings indicated that including plasmids expressing either GM-CSF or LT significantly enhanced the immune responses against multiple 4pox DNA vaccine targets. In general, the adjuvant effect of LT on the 4pox vaccine in mice resulted in a more balanced Th1/Th2 response than GM-CSF, higher antibody binding titers and higher neutralizing antibody titers.

### Mice vaccinated with 4pox/GM or 4pox/LT were protected against lethal challenge with VACV

**Figure 2:**
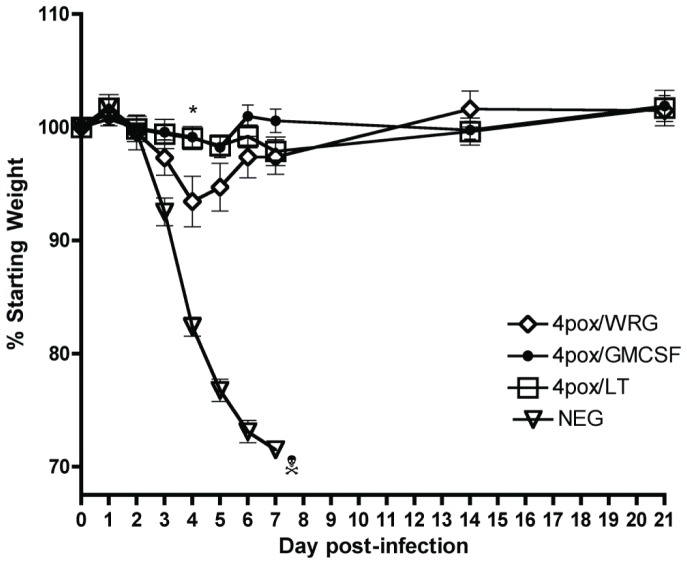
VACV challenge of vaccinated mice. Groups of seven to eight mice vaccinated twice by gene-gun with the indicated DNA vaccine were challenged with 2×10^6^ pfu of VACV strain IHD-J by the i.n. route. Weights of individual mice were monitored for 21 DPI. The percent weights of surviving mice were calculated relative to starting weights (day 0). Mean % weight loss ± standard error of the mean (SEM) were plotted. On day 4 postinfection, there was a significant difference in weight loss between 4pox/WRG versus 4pox/GMCSF (p = 0.0336) or 4pox/LT (p = 0.0384), this point is denoted by an asterisk. Poison symbol indicates all animals in the group became moribund and were euthanized.

To understand how the enhanced immune responses impacted protective efficacy of the 4pox vaccine, 3 weeks after the booster vaccination, the mice were challenged i.n. with 2×10^6^ pfu of VACV strain IHD-J. Weight loss and survival were monitored for 21 days postchallenge (**Fig. 2** **and data not shown**). Animals vaccinated with irrelevant DNA began to lose weight by day 2 and succumbed to infection by day 7. Regardless of whether an adjuvant was added, all 4pox vaccinated animals survived challenge. On day 4, mice vaccinated with the adjuvanted vaccines lost significantly less weight (p<0.05) compared to 4pox/WRG-vaccinated animals. Overall the maximum amount of weight loss for the 4pox vaccinated mice was ∼7.5 g versus ∼3 g for mice receiving the adjuvanted vaccines. By 21 days postchallenge all 4pox vaccinated animals, with or without adjuvant, returned to +/−5% of their starting weight. These findings demonstrated that the 4pox vaccine could produce immune responses capable of protecting mice against a lethal i.n. VACV challenge after a single boost. Furthermore, they showed that addition of GM-CSF or LT could decrease morbidity (p<0.05), as measured by a decrease in weight loss on day 4.

### The 4pox/LT DNA vaccine delivered by ND10 device elicited robust antibody responses in NHPs

**Figure 3:**
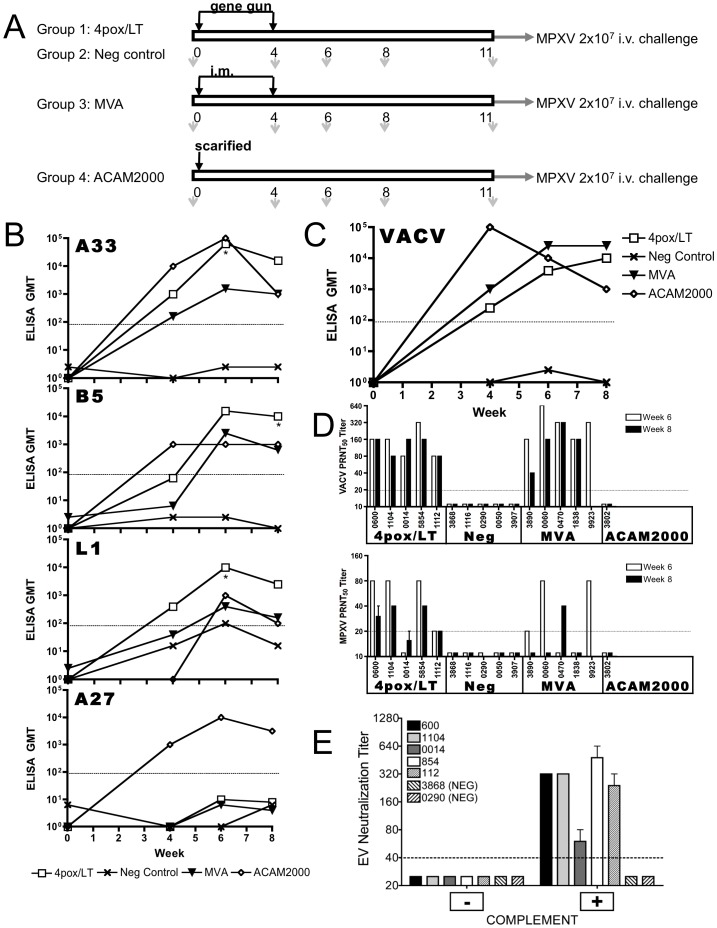
Vaccination of NHP with 4pox, MVA and ACAM2000. **A**. Schematic showing the vaccination protocol. NHPs were vaccinated with the 4pox/LT vaccine or negative control DNA by the ND10 device twice at 4-week intervals. Another group of NHP was intramuscularly vaccinated with MVA (ACAM3000) 1×10^8^ pfu/dose twice at a 4-week interval. One NHP was vaccinated with a single dose of ACAM2000 and served as a positive control. The down arrows indicate bleed dates. **B**. Sera from individually vaccinated NHPs were collected at week 0, 4, 6 and 8 and level of antigen-specific antibodies was examined by protein-ELISA as in Figure 1, with the exception that a monkey secondary was used (1∶2000). Data were plotted as a line graph showing the GMT for each group. **C**. VACV strain WR infected-cell lysate or mock antigen was used as ELISA antigen. Serial 10-fold dilutions of individual NHP serum samples were evaluated by ELISA and the GMT for each group at each time-point were plotted. **D**. VACV- and MPXV-neutralizing antibody titers for weeks 6 and 8 were determined by PRNT as described in Figure 1. Mean PRNT50 titers from two experiments are shown for individual NHP at each time point. Error bars represent mean titers ±SEM. **E**. EEV neutralization. Sera from NHPs vaccinated with 4pox/LT (600, 1104, 0014, 854 and 112) or negative control (3868 and 0290) was diluted twofold starting at a 1∶40. Samples were incubated with fresh EV particles in the presence or absence of complement as indicated. Anti-MV antibodies (anti-L1 MAb-10F5) were included in all samples to remove residual MV. After incubation, EV particles were adsorbed to BSC-1 cell monolayers and incubated for 4 days when they were stained for plaque formation. Titers were determined as described in the material and methods. Each sample was run in duplicate. Mean values ±SD are shown. The limit of detection was a titer of 40 (dashed line).

We were interested in determining if a four-gene DNA vaccine (4pox) adjuvanted by DEI-LT and delivered by a device designed for use in humans (Pfizer's ND10 device) could elicit protective immunity in NHP after a single boost. Moreover, we were interested in comparing this molecular vaccine side-by-side with MVA, a vaccine currently fast-tracked for licensure by the FDA. To evaluate the performance of the 4pox/LT vaccine relative to MVA, groups of five NHPs were vaccinated twice at a 4-week interval with 4pox/LT or MVA. The experimental design is shown in **Fig. 3A**. The 4pox/LT DNA vaccine formulated into ND10 devices was delivered to the skin, and MVA was administered i.m. using a needle and syringe. The 4pox/LT vaccine consisted of the same plasmids used in the mouse experiments mixed 1∶1∶1∶1∶1 and then formulated in the ND10 devices (Pfizer). As negative controls, five NHPs were vaccinated with ND10 devices containing gold without plasmid DNA. As a positive control, a single NHP was vaccinated with the licensed vaccine, ACAM2000. Sera from vaccinated animals were collected on weeks 0, 4, 6 and 8 and evaluated for antibody responses. After a single vaccination (week 4), NHPs vaccinated with 4pox/LT developed antibody responses to L1, A33 and B5, but not against A27, as measured by immunogen-specific ELISA (**Fig. 3B**). After the booster vaccination, the mean antibody titers dramatically increased, including a modestly detectable A27 response in some animals (week 6). The mean antibody titers against A33, B5, and L1 remained essentially the same 1-month after the last vaccination (week 8). MVA-vaccinated animals also developed antibody responses against all four targets by week 8. The mean ELISA titers for weeks 6 and 8 for L1, A33, B5 produced by vaccination with 4pox/LT were >1 log higher than those produced by MVA. Both vaccines resulted in low A27 responses. The 4pox/LT responses to L1, B5 and A33 were statistically significant compared to the MVA vaccinated animals on at least one day as indicated. The NHP vaccinated with ACAM2000 also developed antibody responses against all four targets, including a robust A27 response.

Because the MVA and ACAM2000 vaccines consist of live-virus and encode hundreds of protective and non-protective targets other than L1, A27, B5 and A33, we also examined the antibody responses generated by the vaccine using a VACV-infected-cell lysate ELISA (**Fig. 3C**). In this assay, both ACAM2000 and MVA exhibited robust responses on week 4. The mean MVA response increased on week 6 and remained the same through week 8. The ACAM2000 response peaked on week 4 and dropped two logs by week 8. The 4pox/LT vaccine also produced a response against the whole virus and by week 8. This response was only slightly below that of the MVA response, a ∼0.5 log difference. There were no statistical differences between the responses to infected cell-lysate ELISA for any group. We also detected antibodies against LT in NHPs receiving the 4pox/LT vaccine (**Fig. S1**), indicating the adjuvant itself was immunogenic.

To investigate the levels of MV neutralizing antibodies produced by the vaccines, VACV and MPXV PRNT were performed on sera collected on weeks 6 and 8. Sera from 4pox/LT-vaccinated NHP had VACV PRNT GMTs of 139 and 121 on weeks 6 and 8, respectively (**Fig. 3D**). Similarly, sera from MVA-vaccinated NHP had VACV PRNT GMTs of 278 and 121 on weeks 6 and 8, respectively. Neither the negative control sera nor sera from ACAM2000 neutralized VACV. Sera from vaccinated animals were tested in MPXV PRNT. Overall, there was a marked reduction in neutralizing activity against MPXV compared to VACV for both MVA and 4pox/LT. However, sera from all the 4pox/LT-vaccinated animals neutralized MPXV, with GMTs of 40 and 27 on weeks 6 and 8, respectively. On week 6, only three MVA-vaccinated animals had detectable titers (GMT = 26), and by week 8 only one of four sera had a detectable titer (the week 8 #9923 sample was not available) (**Fig. 3D**). Similar to what was observed in the VACV PRNT, neither the negative control sera nor sera from ACAM2000 neutralized MPXV.

To evaluate the anti-EV functional antibody response, we examined the ability of the sera from vaccinated NHPs to neutralize EV particles in the presence or absence of complement. Pooled sera from vaccinated NHPs were incubated with EV particles in the presence of an MV-neutralizing monoclonal antibody (anti-L1 MAb-10F5). In the absence of complement, none of the samples neutralized EV particles. When complement was added, only the 4pox/LT serum was capable of neutralizing EV particles with a titer of 160 (data not shown). We subsequently tested each of the 4pox/LT-vaccinated animals individually and determined that all five had EV neutralizing antibodies with titers ranging from 60 to 480 (**Fig. 3E**). Together these findings demonstrated that the 4pox/LT vaccine could produce binding antibodies against three of the four target proteins and functional (neutralizing) anti-MV and anti-EV antibodies when delivered to NHP using the ND10 device. Levels of antibodies against the L1, A33, and B5 were highest for the 4pox/LT vaccine group; whereas the overall antibody response to infected-cell lysate were statistically insignificant between the groups.

### The 4pox/LT DNA vaccine protected NHP from lethal MPXV challenge to levels similar to MVA vaccinated animals

The protective efficacy of the 4pox/LT, MVA and ACAM2000 vaccines in NHPs was evaluated by exposing the vaccinated animals to 2×10^7^ pfu of MPXV strain Zaire-79 by the intravenous route (**Fig. 3A**). Five NHPs vaccinated with the negative-control DNA vaccine served as the negative controls. Survival, lesion counts, WBC counts, body temperature and weight were monitored for 28 days.

**Figure 4:**
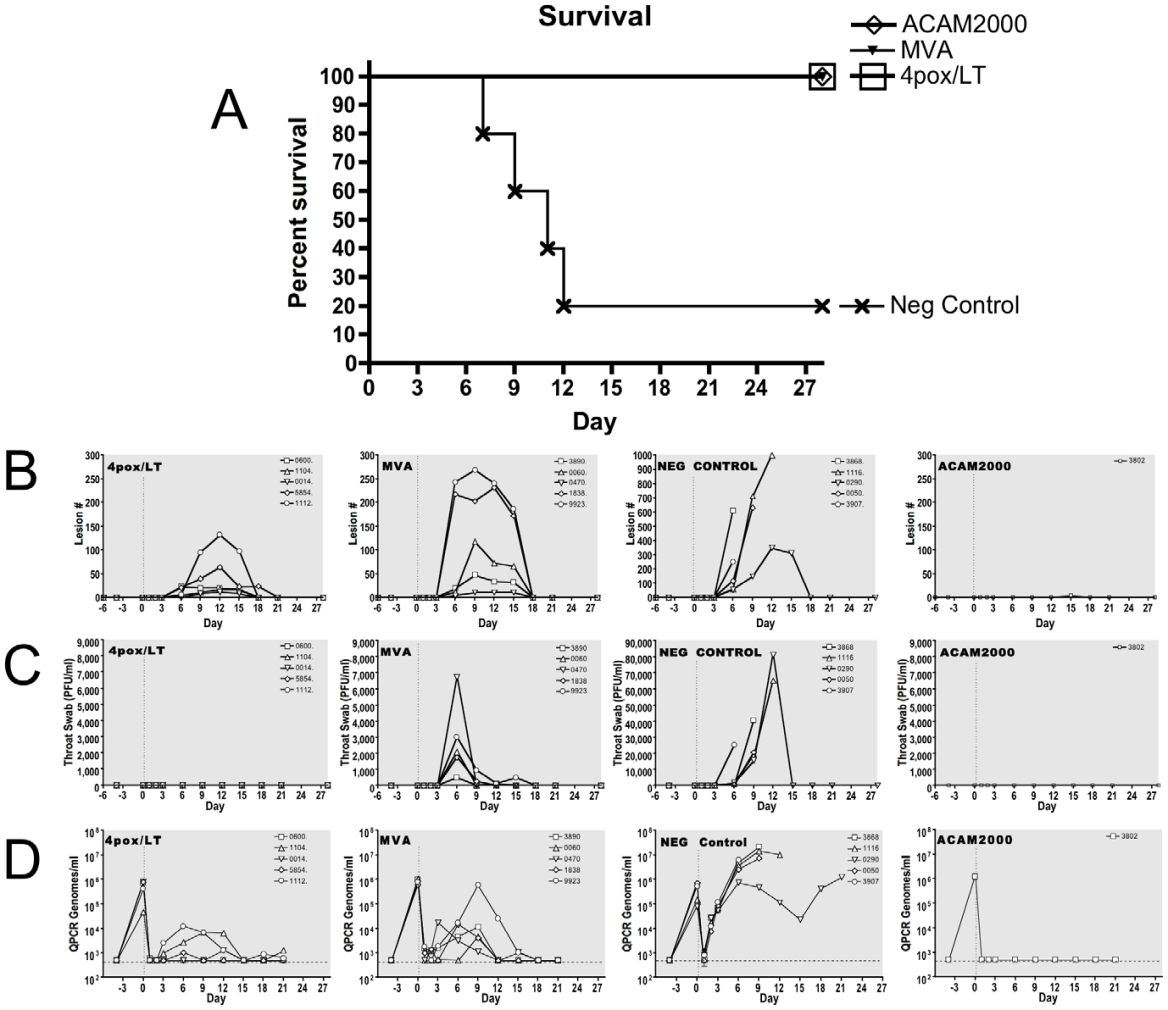
MPXV challenge of vaccinated NHPs. Groups of vaccinated NHPs were challenged with 2×10^7^ pfu of MXPV strain Zaire-79 by the intravenous route. **A.** Survival was monitored out to 28 DPI. Moribund animals were euthanized and the percent survival was plotted. The 4pox/LT and MVA vaccines exhibited a significant level of protection relative to the negative controls as measured by % survival (p-value 0.0145) and survival curve (p-value 0.0399) **B.** Pox lesions on infected NHPs were counted on days 0, 1, 2, 3, 6, 12, 15, 18, 21, and 28. Lesions were individually counted on the hands, feet, torsos, face, mouth arms and legs. The total lesion count per animal from each time-point is plotted. **C.** Shedding of viable virus from the throat was monitored on days 0, 1, 2, 3, 6, 12, 15, 18, 21, and 28 by plaque assay as described in the materials and methods. **D.** MPXV genomes in the whole blood of the animals were determined at the indicated time-points by quantitative PCR. The day 0 blood sample was collected immediately after i.v. injection of the challenge virus. The assay limit was 500 genomes per ml.

All of the NHPs vaccinated with the 4pox/LT, MVA, or ACAM2000 survived challenge; whereas four of the five negative controls became moribund and were euthanized on days 7, 9, 11 and 12 (**Fig. 4A**). Negative control NHPs lost considerably more weight versus 4pox/LT-, MVA- and ACAM2000-vaccinated animals (**Fig. S2A and data not shown**). All of the NHP in this study, including the ACAM2000 control animal, developed at least a few pox lesions (**Fig. 4B**). Pox lesions were first observed on day 6. In negative-control animals that succumbed, the lesion numbers increased until euthanasia. In the single negative control survivor, the lesion number peaked on days 12–15 and then the lesions resolved. On the whole, the NHP vaccinated with 4pox/LT developed fewer lesions compared to the negative controls and these lesions peaked on day 12. Only one animal (#1112) developed severe disease (132 lesions) on day 12. These lesions were localized to the hands and feet, and not the face or other regions. MVA-vaccinated animals also developed fewer lesions compared to the negative controls; however, three of the five NHPs developed severe disease (>100) with lesion counts for two animals >200. Peak lesion numbers are provided in **Table 1**. Peak lesion counts and overall change in lesion count overtime were not statistically different between the 4pox/LT and MVA groups (p = 0.0916); however, when daily lesion counts were compared by ANOVA using the day as a repeated measure, there was a significant difference in the daily lesion counts between the groups (p = 0.0028).

Shedding of infectious virus from the throat/oral cavity was monitored by plaque assay. Negative-control NHPs shed significant amounts of infectious virus beginning on day 6 (**Fig. 4C** and **Table 1**). Titers in these animals ranged from 25,000 pfu/ml to 81,250 pfu/ml. The last day infectious virus was detected was on day 12 in the sole negative control survivor. All five NHPs vaccinated with MVA shed infectious virus, but to a markedly reduced extent compared to the negative control animals. Shedding from MVA-vaccinated animals ranged from 500 pfu/ml to 6,725 pfu/ml and was mostly observed on day 6. There was no infectious virus detected in the throat/oral swabs of the five NHP vaccinated with 4pox/LT. An ANOVA with day as the repeated measure showed a significant difference in the daily throat swab viremia (p = 0.0038), and a significant overall change over time (p<0.0001) between 4pox/LT and MVA groups. The single ACAM2000-vaccinated animal was also negative for infectious virus shedding.

To measure viremia in the blood, samples were evaluated by plaque assay to detect infectious virus, and by quantitative PCR to measure viral genomes. Infectious MPXV was not detected in any of the postexposure samples (limit of detection 500 pfu/ml) (data not shown). However, MPXV genomes were detected. All 16 animals in this experiment exhibited high levels of genome in the blood on day 0 (postinjection) confirming successful intravenous injection of challenge virus (**Fig. 4D**). Genome levels then dropped 2–3 logs 1 day after challenge. In the negative controls, genomes started to increase on day 2 and then peaked between days 6 and 9. The single survivor in the negative control group, #0290, was lesion free and no longer shedding virus by day 18; however, this animal still had detectable levels of genome in the blood (**Fig. 4D**) and an elevated white blood cell count (**Fig. S2B**) on the last day tested (day 21). The NHPs vaccinated with the 4pox/LT vaccine exhibited dramatically reduced viremia relative to the negative controls. None of those animals had titers >10^4^ and 2/5 animals had levels below detection on all but one sample day. NHPs vaccinated with MVA also exhibited dramatically reduced viremia relative to the negative controls. However, four of five of the MVA-vaccinated NHP had genome viremia at ≥10^4^ on at least one sample day. One animal (#0060) had genome levels below detection in all but one sample. An ANOVA with day as the repeated measure showed a significant difference in the daily log_10_-transformed Q-PCR viremia (p = 0.0159) between the 4pox/LT and MVA groups, and a significant overall change over time (p<0.0001). However, the interaction between time and group was not significant (p = 0.3300) indicating the change in Q-PCR genome levels overtime did not differ between groups.

## Discussion

We, and others, have previously tested molecular smallpox vaccines in the NHP MPXV intravenous challenge model [29], [30], [31], [32], [33], [45]. Most studies involved a prime followed by multiple booster vaccinations, including a recent DNA vaccine study that delivered the 4pox immunogens plus four others by both skin and muscle electroporation [33]. Only two studies involved a single boost. In one study, the 4pox targets were formulated into an alphavirus replicon system and the challenge dose was 5×10^6^ pfu of MPXV [31]. In a second study, the 4pox targets were delivered as purified proteins combined with adjuvant and the challenge dose was 2×10^7^ [30]. In both of those studies there was 100% protection against lethal disease and none of the vaccinated animals developed >100 lesions (i.e., no severe disease). Here, we report the first study where a smallpox DNA vaccine delivered as a single boost has been tested in the NHP MPXV intravenous challenge model. Moreover, it is the first side-by-side comparison of any molecular smallpox vaccine against MVA. Our reason for directly comparing the 4pox vaccine with MVA was to determine if a DNA vaccine could elicit protective immunity comparable to an attenuated virus vaccine already in advanced development. Advantages and disadvantages of an effective DNA vaccine versus a protein subunit, viral-vectored, or attenuated live-virus vaccine have been discussed elsewhere [46]. Before performing the direct comparison study in NHP, we sought to maximize the potency of the 4pox vaccine. The L1R, A27L, A33R, and B5R plasmids used in this study all contained open reading frames that were optimized for mammalian expression and mRNA stability (46). In addition, the L1R open reading frame was modified by the addition of an endoplasmic reticulum (ER) retention signal. This modification targeted the L1 protein to the ER resulting in correct disulphide bond formation and protein folding [36]. These improvements resulted in higher levels of neutralizing antibodies after fewer boosts, and overcame previously observed interference when vaccinations involved the co-delivery of combinations of the L1R and A33R plasmids [27].

Until this study, plasmid-encoded adjuvants had not been tested for a capacity to increase the immunogenicity of candidate poxvirus DNA vaccines. Here, we performed experiments in mice that evaluated the feasibility of using plasmid-encoded immunostimulatory molecules (GM-CSF and LT) to enhance the immunogenicity of the 4pox DNA vaccine. GM-CSF was first shown by Xiang, Z. et al to augment a rabies virus DNA vaccine [47]. It has since been reported to be effective at enhancing immune responses against a variety of other DNA vaccines, including enterotoxigenic *E. coli* in pigs and herpes simplex virus-2 in mice [48], [49]. GM-CSF functions as a potent molecular adjuvant by recruiting and activating antigen-presenting cells (APCs) to the area where vaccine target proteins are being expressed [50]. LT is among the most potent adjuvants known, with activity as low as 2.5 ng/dose [51]. It consists of two subunits, the A subunit contains the ADP ribosyltransferase activity (e.g., toxin activity), and the B subunit is involved in targeting and entering eukaroyotic cells (for review see [52]). The mechanism by which LT enhances immune responses is not clear, but may involve the ability of this family of toxins to activate dendritic cells [53]. While the use of wild type LT is not possible for many vaccine platforms (e.g., protein) due to toxicity [54], [55], [56], Arrington, J., et al, were the first to show that wild type LT functions as a safe DNA vaccine adjuvant [37]. Haynes, JR., et al demonstrated that A and B subunits of LT encoded on the same plasmid can form a fully functional toxin *in vitro* and function as a safe DNA vaccine adjuvant *in vivo* [38]. We found that both GM-CSF- and LT-encoding plasmids significantly improved the antibody responses against three of the four target immunogens, and both resulted in a significant decrease in weight loss on day 4 postchallenge (**Fig. 2**). The LT plasmid was selected for use in the NHP study for multiple reasons. First, mice vaccinated with 4pox/LT had higher, albeit not significantly higher, antibody GMT than mice vaccinated with 4pox/GM-CSF in the four immunogen-specific ELISA and in the PRNT. Second, mice vaccinated with 4pox/LT exhibited an IgG1/IgG2a ratio of 1 for all four immunogen-specific ELISA, suggesting a more balanced immune response. Third, unlike GM-CSF, LT is not a host protein and does not require a species-specific receptor for immunostimulatory activity. This is an important attribute because it allows the use of the same plasmid adjuvant in divergent species. A different GM-CSF plasmid would be needed for vaccine testing in mice, rabbits, nonhuman primates, and humans. Furthermore, orthopoxviruses currently infect agriculturally important animals throughout the world [2], [57], [58], raising the potential that this vaccine may be used not only in humans, but also in other species.

As a DNA vaccine delivery technology, we decided to use a hand-held gene gun (ND10) that was developed for clinical use. This device was chosen because 1) most of our early work involved the use of a research gene gun (particle mediated epidermal delivery device) so we had reason to believe that the 4pox targets would be immunogenic when delivered by the ND10, 2) the ND10 uses two-three orders of magnitude less DNA than other technologies such as muscle electroporation, and 3) we had recently conducted a Phase 1 trial of a hantavirus DNA vaccine delivered using the ND10 device and the data demonstrated that the device was safe, tolerable, and capable of eliciting high-titer neutralizing antibodies in humans [59]. Less satisfactory results were obtained using the ND10 to deliver a candidate influenza vaccine [60]. Our findings in the current study support the efficacy of the ND10; however, the inconsistency of the ND10 results in the clinic indicate that modifications of the technology might be required. In addition to gene gun, other devices and methods to deliver DNA vaccines continue to evolve. For example, a DNA vaccine comprised of the 4pox targets plus four additional targets delivered by intradermal and intramuscular needle-injection followed by electroporation was recently tested in NHP [33]. In that study, NHP were protected against a 2×10^7^ challenge after three vaccinations (days 0, 28, 56). Other vaccine delivery technologies that are already FDA 510(k) cleared for use in the delivery of vaccines, such as needle-free jet-injection, are currently being tested in animals for efficacy in delivering DNA vaccines (Kishimori, J and Hooper, J.W., unpublished data).

In this study we did not evaluate cell-mediated immune responses to the vaccines. Rather we focused on antibody responses, and particularly neutralizing antibody responses directed at both MV and EV. A critical role for antibody in vaccine-induced protection against orthopoxviruses has been reported extensively (for a review see 22; see also 19, 21)). For example, Edghill-Smith et al. demonstrated that vaccinia immune globulin was sufficient to protect NHP from lethal MPXV challenge; whereas CD8+ T cells were dispensable [19]. More recently, we reported that serum from a NHP vaccinated with the 4pox DNA vaccine was sufficient to confer protection in passive transfer studies in mice [41]. Indeed, a critical role for cytotoxic T-cells in protection after 4pox vaccination has not been shown experimentally to date.

The NHP experiment performed herein revealed that the ND10 could effectively deliver three of the four pox immunogens. Antibodies to A27 after vaccination with the 4pox/LT were not detected by ELISA. Interestingly, MVA also failed to elicit a detectable anti-A27 antibody response in this study and others have reported low anti-A27 responses in humans vaccinated with MVA ([61]). We ruled out assay error because sera from the NHP vaccinated with ACAM2000 developed a potent A27 response. Our findings, among others, have found that the A27 response after vaccination with a molecular vaccine can vary widely depending on the delivery technology and the animal species [30], [31], [62], [63]. Additional research will be needed to solve the A27 immunogenicity issue or, alternatively, the A27L component of the vaccine could be replaced with another immunogen known to elicit anti-MV neutralizing antibodies (e.g., D8L or H3L) [64], [65], [66]. This would still allow redundancy in MV targeting, which is the reason for including two MV and two EV immunogens in the 4pox vaccine. Despite the poor response to A27, the 4pox/LT DNA vaccine elicited potent responses to L1, B5, and A33 resulting in high titers of functional antibodies as measured by MV and EV neutralization assays. In our hands, the MVA vaccine elicited MV neutralizing antibodies, but did not elicit EV neutralizing antibodies. This was a notable difference detected between the 4pox/LT and MVA vaccines and is a possible explanation for the significant differences in protection discussed below. Others have reported that EV neutralizing antibodies were associated with the highest levels of protection in NHP vaccinated with purified L1, A27, B5, and A33 [30].

When compared directly, both the 4pox/LT and MVA vaccines completely protected macaques against lethal monkeypox. There were three significant differences between the levels of protection against disease afforded by 4pox/LT versus MVA. First, NHP vaccinated with 4pox/LT presented numbers of lesions on the skin over-time that were significantly lower than those presented NHP vaccinated with MVA. Only one of five 4pox/LT-vaccinated animals developed severe disease (>100 lesions) compared to 3/5 MVA-vaccinated NHP. Second, the NHP vaccinated with 4pox/LT developed significantly less viremia over-time than the NHP vaccinated with MVA. Third, and most significant, the NHP vaccinated with 4pox/LT did not have detectable levels of infectious virus in oral secretions, whereas infectious virus was detected in the oral secretions of all five NHP vaccinated with MVA. To our knowledge, this is the first NHP study where a molecular smallpox vaccine has prevented the shedding of infectious virus in the oral cavity in all vaccinated animals. Interestingly, the 4pox DNA vaccine delivered by VEE-replicon prevented blood viremia, as measured by Q-PCR, in all 10 vaccinated NHP, but all animals had detectable levels of virus genome in their oral swabs [31]. In that study, assays for infectious virus in oral swabs were not performed. The inability of the MVA vaccine to prevent throat shedding has been observed previously in an inter-tracheal MPXV/NHP challenge model [67]. It should be noted that, although MVA did not completely eliminate shedding of infectious virus, the level of virus in the oral secretions was significantly reduced relative to the level in the negative control animals. Relevance of this low level of virus shedding in oral secretions remains to be determined, especially since both the intravenous challenge and inter-tracheal MPXV/NHP challenge models involve high-dose challenges by artificial routes. The aerosol RBXV/rabbit challenge model [68] may be better suited to fully characterize the ability or inability of MVA to prevent viral shedding.

Our finding that the 4pox DNA vaccine is comparable to MVA when delivered as a prime followed by a single boost in NHP argues that this subunit vaccine could be used for certain indications targeted by MVA-based vaccines such as IMVAMUNE® or ACAM3000. For example, these vaccines could be used to vaccinate persons who are contraindicated for ACAM2000, or to prime vaccinees to improve the safety of a subsequent booster vaccination with ACAM2000 [69], [70]. The 4pox vaccine might be better suited than MVA for other indications, such as boosting persons previously vaccinated with ACAM2000. This is because, unlike MVA, preexisting anti-VACV immunity would not neutralize a DNA vaccine. On the other hand, data from an NHP study demonstrating that a single high-dose vaccination with MVA followed by a rapid MPXV challenge elicited a more rapid antibody response and was more protective than ACAM2000 indicates that MVA might be better suited than ACAM2000 (or a DNA vaccine) as an emergency vaccine [71]. In our study, the ELISA and PRNT responses after a single MVA vaccination were approximately the same or lower than those achieved with 4pox/LT or ACAM2000 (**Fig. 3**). However, our first time-point was 4 weeks after the first vaccination, so we would not have detected a more rapid response at an earlier time-point. We have not tested the 4pox DNA vaccine in an emergency vaccination (postexposure) experiment; however, it is unlikely that the adaptive immune response would be rapid or potent enough to confer protection. The role that vaccine-induced innate immunity plays in postexposure protection is not known, but a priori evidence suggests it must contribute to protection. Whether the innate response after a DNA vaccine could elicit the level or quality of innate immunity (e.g., type 1 interferon) than that elicited by a high-dose injection of MVA, is not known.

Unfortunately, safety concerns associated with the licensed replicating VACV-based vaccines (e.g. ACAM2000) limit their use as a pretreatment in the general population. Arguments that the cost of developing and stockpiling future generation orthopoxvirus pretreatments is prohibitive [72] might be tempered if those costs were dramatically reduced by fundamentally changing the vaccine technology and by exploring unique paths to licensure. Molecular vaccine technologies, and DNA vaccine technology in particular, continue to evolve in terms of potency and practicality and could prove to be a modern alternative to the virus-based vaccine platforms of the past. Novel paths to licensure could include demonstrating that the product is reasonably likely to confer a clinical benefit, rather than demonstrating that the product is equivalent to a historical vaccine known to protect against smallpox. For example, an exceedingly safe product that is clinically proven to safely boost levels of MV and EV neutralizing antibodies in persons vaccinated years earlier with Dryvax or ACAM2000 might be argued to be reasonably likely to confer a clinical benefit to the recipients (i.e., increased resistance to infection and disease caused by pathogenic orthopoxviruses). A safe and deployed pretreatment strategy would reduce our reliance on post-event emergency response capabilities.

## Supporting Information

Figure S1 **Detection of anti-LT responses in vaccinated NHPs.** COS cell monolayers (70 to 80% confluent) were transiently transfected with pPJV2012(DEI-LT) (black line) or an empty vector (grey area) in T25 flasks with indicated constructs using Fugene 6. This plasmid encodes encodes the LT toxin A and B units. Transfected cells were incubated at 37°C for 48 h, trypsinized, and washed once with EMEM. After the wash, ∼1×10^6^ cells were transferred to 14-ml polystyrene tubes. Cells were fixed (30 min) using fixation buffer (BD Biosciences) and then permeabilized in wash/permeabilization buffer (BD Biosciences) according to the manufacturer's directions. Cells were incubated with serum from 4pox/LT or sham vaccinated animals (1∶100) for 1 h at room temperature in wash/permeabilization buffer. After incubation with the primary antibody, cells were pelleted by low speed centrifugation for 1 min and washed twice with wash/permeabilization buffer. Cells were next incubated with an anti-monkey FITC antibody (Invitrogen) (1∶500) for 30 min at room temperature. After incubation with the secondary antibody, cells were pelleted by centrifugation at 750× *g* for 3 min. Washed cells were resuspended in 1 ml of FACS buffer (PBS, 5% FBS, and 0.1% sodium azide). Flow cytometry was performed on a FACSCalibur flow cytometer (Becton Dickinson, San Jose, CA). Data were collected and analyzed using FlowJo software (Tree Star Inc., Ashland, OR). A total of 10,000 cells were analyzed for each sample.
(TIF)

Figure S2 **Further analysis of vaccinated NHP challenged with MPXV.** **A**. Weight loss in 4pox/LT or sham vaccinated NHPs was monitored at the initiated time points. Weight loss data are displayed as the % weight loss from the starting values determined 6 days before to challenge. **B**. Total white blood cell counts (WBC) were determined at the indicated time points for each group. WBC values were determined from blood samples collected in tubes containing EDTA, using a laser-based hematologic analyzer (Coulter Electronics, Hialeah, FL) as per the manufacturer's protocol. Mean values were plotted and displayed. The dashed lines indicate the normal high and low values for NHPs.
(TIF)
