# Supplementary figures and images for: Side-by-Side Comparison of Gene-Based Smallpox Vaccine with MVA in Nonhuman Primates
Source: PLoS One. 2012 Jul 31;7(7):e42353. doi: 10.1371/journal.pone.0042353 (PMC3409187; doi:10.1371/journal.pone.0042353)

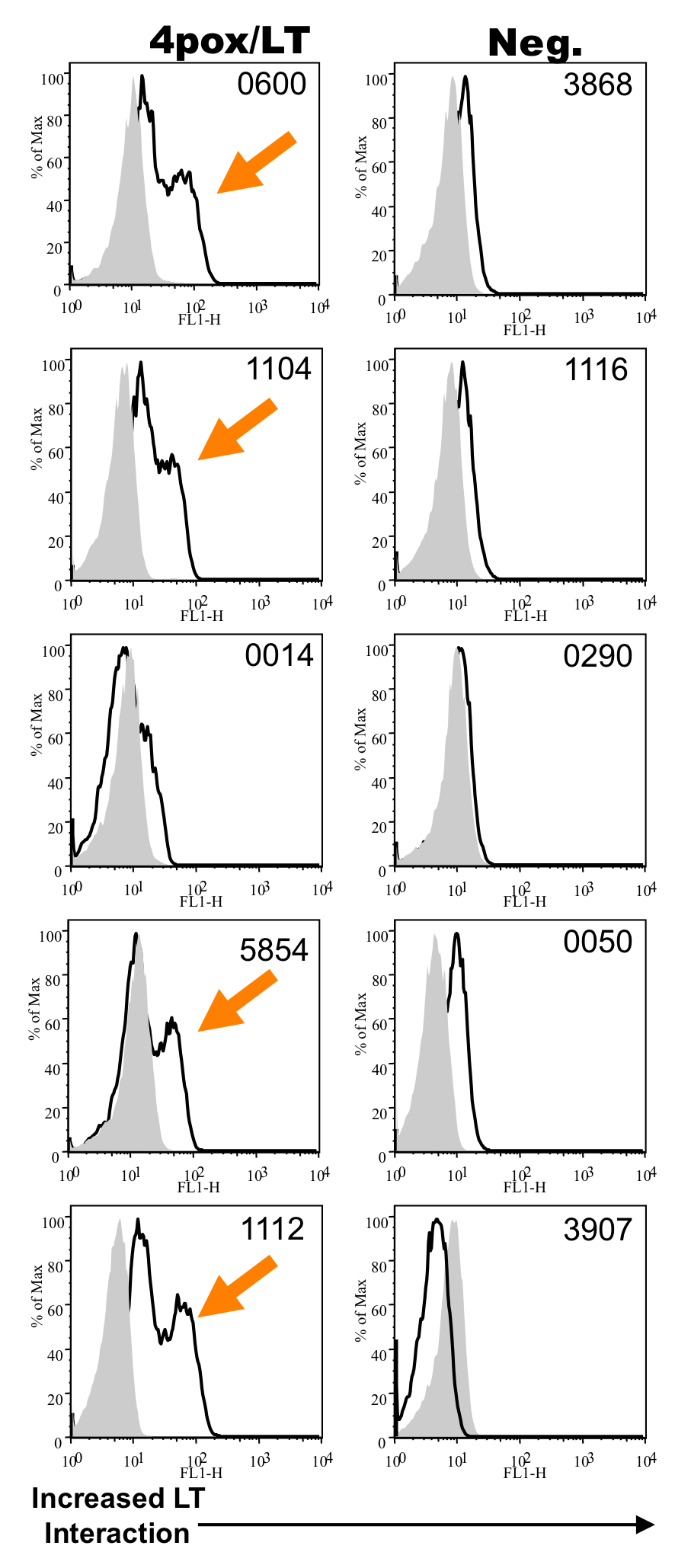

Supplement: Figure S1 — Detection of anti-LT responses in vaccinated NHPs. COS cell monolayers (70 to 80% confluent) were transiently transfected with pPJV2012(DEI-LT) (black line) or an empty vector (grey area) in T25 flasks with indicated constructs using Fugene 6. This plasmid encodes encodes the LT toxin A and B units. Transfected cells were incubated at 37°C for 48 h, trypsinized, and washed once with EMEM. After the wash, ∼1×10^6^ cells were transferred to 14-ml polystyrene tubes. Cells were fixed (30 min) using fixation buffer (BD Biosciences) and then permeabilized in wash/permeabilization buffer (BD Biosciences) according to the manufacturer's directions. Cells were incubated with serum from 4pox/LT or sham vaccinated animals (1∶100) for 1 h at room temperature in wash/permeabilization buffer. After incubation with the primary antibody, cells were pelleted by low speed centrifugation for 1 min and washed twice with wash/permeabilization buffer. Cells were next incubated with an anti-monkey FITC antibody (Invitrogen) (1∶500) for 30 min at room temperature. After incubation with the secondary antibody, cells were pelleted by centrifugation at 750× g for 3 min. Washed cells were resuspended in 1 ml of FACS buffer (PBS, 5% FBS, and 0.1% sodium azide). Flow cytometry was performed on a FACSCalibur flow cytometer (Becton Dickinson, San Jose, CA). Data were collected and analyzed using FlowJo software (Tree Star Inc., Ashland, OR). A total of 10,000 cells were analyzed for each sample. (TIF) [file pone.0042353.s001.tif]

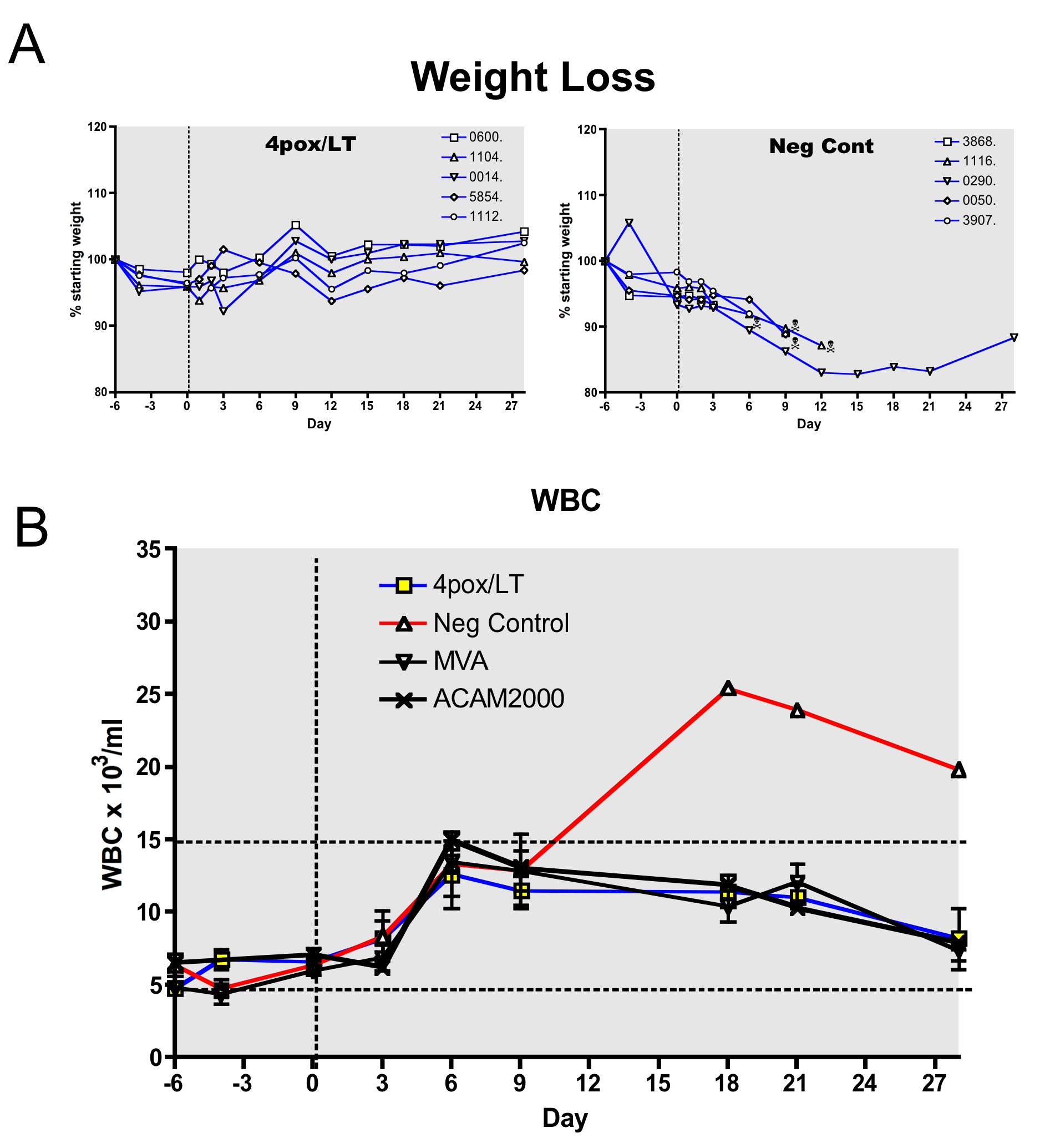

Supplement: Figure S2 — Further analysis of vaccinated NHP challenged with MPXV. A. Weight loss in 4pox/LT or sham vaccinated NHPs was monitored at the initiated time points. Weight loss data are displayed as the % weight loss from the starting values determined 6 days before to challenge. B. Total white blood cell counts (WBC) were determined at the indicated time points for each group. WBC values were determined from blood samples collected in tubes containing EDTA, using a laser-based hematologic analyzer (Coulter Electronics, Hialeah, FL) as per the manufacturer's protocol. Mean values were plotted and displayed. The dashed lines indicate the normal high and low values for NHPs. (TIF) [file pone.0042353.s002.tif]
